# New solution of the partial differential equation of the grain groove profile problem in the case of evaporation/condensation

**DOI:** 10.1038/s41598-019-46537-6

**Published:** 2019-07-12

**Authors:** Tayssir Hamieh, Zoubir Khatir, Ali Ibrahim

**Affiliations:** 1 Systèmes et Applications des Technologies de l’information et de l’Energie (SATIE), Institut français des sciences et technologies des transports, de l’aménagement et des réseaux (IFSTTAR), 25 Allée des Marronniers, 78000 Versailles, France; 2 Laboratory of Materials, Catalysis, Environment and Analytical Methods (MCEMA) and LEADDER Laboratory, Faculty of Sciences and EDST, Lebanese University, Hariri Campus, Hadath, Beirut Lebanon

**Keywords:** Energy science and technology, Materials science, Applied mathematics

## Abstract

This paper constitutes a new contribution on the resolution of Mullins problem in the case of the evaporation-condensation and gives an exact and explicit solution of the second partial differential equation relative to the geometric profile of the grain boundary grooving. New analytical expressions of the solution, the groove profile, the derivative and the groove deep were obtained:$$\begin{array}{c}y{\boldsymbol{(}}x{\boldsymbol{,}}t{\boldsymbol{)}}{\boldsymbol{=}}{\boldsymbol{-}}\,\sqrt{\pi Ct}\,sin\,\theta \,{\boldsymbol{[}}erfc{\boldsymbol{(}}\frac{x}{{\bf{2}}\sqrt{Ct}}{\boldsymbol{)}}{\boldsymbol{+}}{\boldsymbol{\sum }}_{n{\boldsymbol{=}}{\bf{1}}}^{\infty }\,\frac{{\boldsymbol{(}}{\bf{2}}n{\boldsymbol{)}}{\boldsymbol{!}}}{{{\boldsymbol{(}}n{\boldsymbol{!}}{\boldsymbol{)}}}^{{\bf{2}}}{{\bf{2}}}^{{\bf{2}}n}\,\sqrt{{\bf{3}}n}}\,si{n}^{{\bf{2}}n}\theta \,{\boldsymbol{(}}erfc{\boldsymbol{(}}\frac{x\sqrt{{\bf{3}}n}}{{\bf{2}}\sqrt{ct}}{\boldsymbol{)}}{\boldsymbol{)}}{\boldsymbol{]}}\\ y{\boldsymbol{{\prime} }}{\boldsymbol{(}}x{\boldsymbol{,}}t{\boldsymbol{)}}{\boldsymbol{=}}{\boldsymbol{+}}\,\frac{\sin \,\theta }{\sqrt{{e}^{{x}^{{\bf{2}}}{\boldsymbol{/}}{\boldsymbol{(}}{\bf{2}}ct{\boldsymbol{)}}}{\boldsymbol{-}}si{n}^{{\bf{2}}}\theta }}\,{\rm{and}}\,{\varepsilon }_{{\bf{0}}}{\boldsymbol{(}}\theta {\boldsymbol{)}}{\boldsymbol{=}}\sqrt{\pi ct}\,\sin \,\theta \,{\boldsymbol{[}}1{\boldsymbol{+}}{\boldsymbol{\sum }}_{n{\boldsymbol{=}}1}^{\infty }\frac{{\boldsymbol{(}}{\bf{2}}n{\boldsymbol{)}}{\boldsymbol{!}}}{{{\boldsymbol{(}}n{\boldsymbol{!}}{\boldsymbol{)}}}^{{\bf{2}}}{{\bf{2}}}^{2n}\sqrt{3n}}si{n}^{2n}\theta {\boldsymbol{]}}\end{array}$$It was proved that the found solution gave more accurate results relative to those obtained by Mullins that neglected the first derivative (|*y*′| ≪ 1) relative to 1. The results obtained by this new solution can be advantageously used to give more precise solution of the general problem when combining the two phenomena relative to the evaporation-condensation and the surface diffusion in thin polycrystalline films.

## Introduction

**Figure 1:**
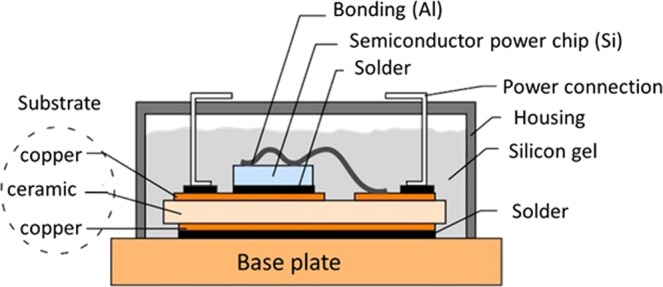
High power IGBT module.

The use of power electronics has increased in recent years. This is due to increased electrification in an economic and social context to reduce energy consumption and greenhouse gas emissions. The size, weight and cost of the converters have decreased, mainly in the field of electronic switches. Power electronic modules are key elements in the chain of power conversion. The application areas include aerospace, aviation, railway, electrical distribution, automotive, home automation, oil industry. These modules constitute an assembly of various materials (Fig. 1).

Generally, the power chips are carried on a ceramic substrate which must ensure good electrical insulation and good thermal conduction. This substrate is also welded on a sole to be cooled.

**Figure 2:**
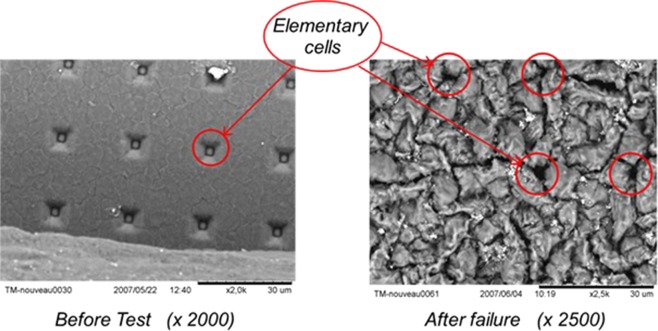
Topside metallization: before and after aging.

**Figure 3:**
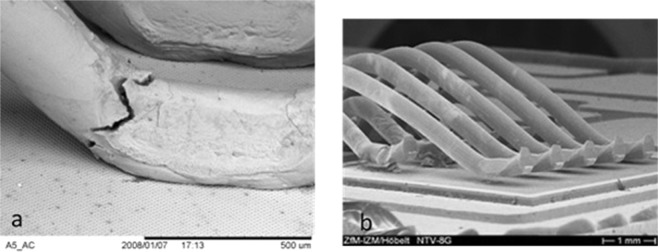
(**a**) Heel cracks, (**b**) Lift off.

There is a diversity of assembly technologies. This includes materials and process for insulation or passivation, interconnections, and die attach. The most common topside interconnections in power semiconductor devices, consisting of the metallization and the wire bonds, are subjected in operation to high functional stresses. This is the result of an important difference between the coefficients of thermal expansion (CTE) of the materials in contact: metallization and wire bonds (aluminum) and dies (silicon). The metallization layer (around 5 μm) deposited on the chips becomes a lot more distorted than the silicon with temperature, leading to high tensile and compressive stresses and thus to large inelastic strains^1^. It has been reported that two main types of degradation can take place in the topside of power chips under the effect of thermomechanical cycles: metallization reconstruction (Fig. 2) and degradation of bonding contacts (Fig. 3)^1–3^. The last one may itself be either heel-cracks or cracks propagation followed by lift-off^4^. Various works have been conducted to propose scenarios of degradation mechanisms using thermal and power cycling tests^5–7^. Although it is quite clear that the wire-bond lift-off contributes mainly to the module failure^8^, this link is not obvious with the metallization degradation^6^.

It is observed that the first phase of aging is classically associated with the reconstruction of the metallization and the degradation of the bonding contact. The end of life is rather characterized by bond-wire heel-cracks and lift-off^9^.

We make the assumption that during cyclic aging, it is a progressive effect of condensation-evaporation that occurs and the film is structurally degraded by grooving. The scenario of this degradation is not clarified yet and it is the purpose of this paper to make a contribution on this point with a better understanding of the effects of stress parameters on the degradation of contacts between metallization and bond wire. It consists in a new mathematical solution of the formation of grain boundary grooving in polycrystalline thin films and in its comparison with other solutions given by Mullins *et al*.^10–16^, Hackney^17^, Broadbridge^18^, Zhang and Wong^19^ and Bouville *et al*.^20,21^.

## Study Problematic

The problem of the thermal degradation and the formation of grain boundary grooving in polycrystalline thin films, was largely studied, analyzed and commented^10–13,19–21^. Mullins^10^ studied in 1957 the thermal effect on the profile of the grain boundary grooving. Other scientists were also interested in the development of this phenomenon^13–21^.

Mullins developed the two cases of the evaporation-condensation and the surface diffusion by formulating in both cases the mathematical problem of the partial differential equation governing the geometric profile of the grain boundary grooving^10–12^.

The method consisted in using the general relation of the curvature *R* at any point (*x*, *t*) of the profile, where *y* = *y*(*x*, *t*) and *R* is a function of the two first derivatives *y*′ and *y″* as a function of *x*. All mathematical developments proposed by Mullins^10–12^ and Mullins *et al*.^13–16^ were based on the approximation given by |*y*′| ≪ 1.

An exact solution was proposed by Broadbridge^18^, based on the Fujita research works^22–24^. However, the proposed solution is not an explicit solution and very complicated to be used directly. To obtain the solution suggested by Broadbridge, it is necessary to determine three parameters Θ_*_, ${\epsilon }$ and *θ*_*m*_ that have to be calculated numerically by solving the found transcendental equation and the integral equation (Equations 21 and 23 of Broadbridge^18^). On the other hand, Broadbridge^18^, and Fujita did not obtain any explicit expression of the solution *y*(*x*, *t*), they only obtained a complicated equation between intermediate variable function of (*x*, *y*) and the first derivative *y*′ (*x*).

The study of the effects of stress parameters on the degradation mechanisms of the top side interconnections will allow to a good understanding of this phenomena. We are interested in this paper, in the mathematical development of a new solution of the problem of the evaporation-condensation by deriving easier and more compact formulas that can be directly used by the scientific community that is interested in this problem type. Explicit equation giving the solution of the general Mullins problem *y*(*x*, *t*) was obtained as a function of *x* and *t*. The new solution will be compared to the Mullins approximated solution^10^.

## Formulation of the Problem

It was observed that a two dimensional metallic film remains flat for any temperatures and for a very long time. When the temperature increases, the metal atoms move causing grooves at the grain boundary surface. The metallic atoms can diffuse at the surface or in the volume. Atoms can also evaporate into the vapor phase or condensate. Mullins^10^ developed the grooving process produced in solid surfaces. Grain-boundary migration controls the growth and shrinkage of crystalline grains and is important in materials synthesis and processing. A grain boundary ending at a free surface forms a groove at the tip, which affects its migration^19^. In polycrystalline thin films, grain boundary grooving through the thickness of the film is a common failure mode that strongly affects their properties. The grooving forms and develops at the point of intersection when the grain boundary ends at a free boundary in order to reduce the total free energy^25^.

Stone *et al*.^25^ studied the surface grooves at grain boundaries that intersect a planar surface for the case that the evolution occurs below the thermodynamic roughening transition by evaporation–condensation processes. They described the resulting groove profile by a nonlinear ordinary differential equation and gave an approximate analytical solution to the nonlinear. However, these authors^21^ as well as Mullins did not obtain an exact solution.

The method developed by Stone *et al*.^25^ is only valid for groove slopes sufficiently shallow that the entire sloping wall is composed of a step train with intervening terraces of the facet orientation. Mullins supposed that all slope tangents of the profile are neglected relatively to 1 (|*y*′| ≪ 1). Both cases developed by Mullins and Stone did not propose an exact solution

**Figure 4:**
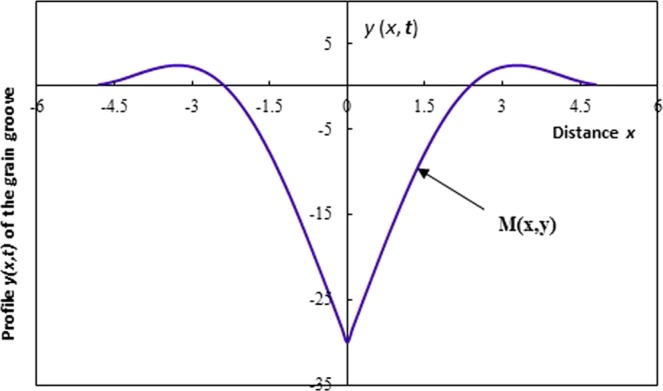
Profile *y*(*x*, *t*) of the grain boundary groove.

As example, we give on Fig. 4 the symmetric profile of grain boundary grooving of a thin film.

It was experimentally shown^26–35^ that when thin polycrystalline films are annealed, they tend to break up by the formation and growth of grooves. Smaller and larger holes are usually located at the intersection points of three-grain boundaries^36^. The break up in thin films of some metals as copper on sapphire was initiated at processing defects in the film^34^, in contrast of cavities found at grain boundaries on zirconia on alumina polycrystalline films^37^.

Let us consider *x* and *y*(*x*, *t*) the spatial coordinates of any point of the groove profile and *t* the time. On Fig. 4, we give the general profile *y*(*x*, *t*) of the grain boundary groove in metal polycrystal.

Galina and Fradkov^38^ studied the problem of the grain boundary hole development by assuming that there is evaporation/condensation as the transport mechanism rather than surface diffusion as generally supposed.

Mullins studied the thermal grooving mechanisms relative to the evaporation/condensation and surface diffusion phenomena^10^. Mullins supposed for a polycrystalline solid at equilibrium, a symmetric grain boundary groove profile and then the ratio of grain boundary energy per unit area, *γ*_*GB*_, to surface energy per unit area, *γ*_*SV*_, is related to the groove angle, *θ* by the following relationship:

$$\frac{{\gamma }_{GB}}{2{\gamma }_{SV}}=Sin\,\theta $$

Note that *tanθ* is equal to the slope of the profile *y*(*x*, *t*) at *x* = 0.

When studying the evolution of grain boundary groove profiles in the cases of the evaporation/condensation and surface diffusion, Mullins^10^ assumed that: (1) the surface diffusivity and the surface energy, *γ*_*SV*_, were independent of the crystallographic orientation of the adjacent grains and (2) the tangent of the groove root angle, γ, is small compared to unity. Mullins also supposed an isotropic material. The assumption (tan*θ* ≪ 1) was used by Mullins to simplify the study of the mathematical partial differential equation. The polycrystalline metal was supposed (3) in quasi-equilibrium with its vapor. The interface properties doesn’t depend on the orientation relative to the adjacent crystals. The grooving process was described by Mullins using the macroscopic concepts (4) of surface curvature and surface free energy. The matter flow (5) is neglected out of the grain surface boundary.

We propose in this paper to study the grain boundary groove profiles in polycrystalline metal and to give an analytical solution relative to the only case of evaporation/condensation, more precise than of the solution found by Mullins^10^ that supposed very small slops for all x values.

By using the notion of curvature *c* at any point M(*x*; *y*(*x*, *t*)) given by the following relation:

1 $$c=\frac{1}{R}=-\frac{y{\prime \prime} (x)}{{[1+y{(x)}^{2}]}^{\frac{3}{2}}}$$

where *R* is the curvature radius at point M, $y{\prime} (x)=\frac{\partial y}{\partial x}$ and $\,{\prime \prime} (x)=\frac{{\partial }^{2}y}{\partial {x}^{2}}$.

The mathematical equation governing the evaporation-condensation problem will be written here as:

2 $$\frac{\partial y}{\partial t}=C(T)\frac{y{\prime \prime} (x)}{(1+y{\prime} {(x)}^{2})}$$

where the parameter *C*(*T*) (equal to Mullins parameter *A* used in equation 5 of ref.^10^) depends on the temperature *T*. It is given by:

3 $$C(T)=\frac{{P}_{0}(T)\gamma {\omega }^{2}}{{(2\pi m)}^{1/2}{(kT)}^{3/2}}$$

where *γ* is the isotropic surface energy, *P*_0_(*T*) the vapor pressure at temperature *T* in equilibrium with the plane surface of the metal characterized by a curvature *c* = 0, *ω* is the atomic volume, *m* is molecular mass and *k* is the Boltzmann constant. Mullins^10^ supposed that the coefficient of evaporation is equal to the unit.

## Mullins Approximation

To resolve this differential equation, Mullins was constraint to suppose that |*y*′| ≪ 1 that means that the slope *y*′(*x*, *t*) at any point of the curve *y*(*x*, *t*) is very small behind 1 and can be neglected. Equation (2) can be written as:

4 $$\frac{\partial y}{\partial t}=C(T)y{\prime \prime} (x)$$

With the boundary conditions:

5 $$\{\begin{array}{l}y(x,0)=0\\ y{\prime} (0,t)=\,\tan \,\theta =m\end{array}$$

This problem is well-known in the conduction of heat in solids. It can be resolved by the following variable change:

6 $$u=\frac{x}{2\sqrt{Ct}}$$

and one obtains the derivatives of u as a function of *x* and *t*:

7 $$\{\begin{array}{l}\frac{\partial u}{\partial x}=\frac{u}{x}\\ \frac{\partial u}{\partial t}=-\,2C\frac{{u}^{3}}{{x}^{2}}\end{array}$$

Now, using $\frac{\partial y}{\partial t}=\frac{\partial y}{\partial u}\frac{\partial u}{\partial t}$ and $\frac{\partial y}{\partial x}=\frac{\partial y}{\partial u}\frac{\partial u}{\partial x}$, one obtains the following derivatives:

8 $$\frac{\partial y}{\partial t}=-\,2C\frac{{u}^{3}}{{x}^{2}}\frac{\partial y}{\partial u}$$

9 $$y{\prime} (x)=\frac{\partial y}{\partial x}=\frac{u}{x}\frac{\partial y}{\partial u}$$

10 $$y{\prime \prime} (x)=\frac{{\partial }^{2}y}{\partial {x}^{2}}={(\frac{u}{x})}^{2}\frac{{\partial }^{2}y}{\partial {u}^{2}}$$

Then, equation (4) becomes as a function of u:

11 $$\frac{{\partial }^{2}y}{\partial {u}^{2}}=-\,2u\frac{\partial y}{\partial u}$$

and

12 $$\frac{y{\prime \prime} (u)}{y{\prime} (u)}=-\,2u$$

The solution of differential equation (12) is given by:

$$y{\prime} (u)=A{e}^{-{u}^{2}}$$

With *A* a constant of the problem.

Knowing that $\frac{\partial y}{\partial u}=\frac{\partial y}{\partial x}\frac{\partial x}{\partial u}$, we obtain:

13 $$\frac{\partial y}{\partial u}=2\sqrt{Ct}\frac{\partial y}{\partial x}$$

With the condition boundary $y{\prime} (0,t)=m$, one obtains $A=2m\sqrt{Ct}$. Using the other condition boundary *y* (*x*, 0) = 0, the solution of the differential equation (13) becomes:

14 $$y=2m\sqrt{Ct}{\int }_{0}^{u}\,{e}^{-{u}^{2}}du+Cst,\,u=\frac{x}{2\sqrt{Ct}}$$

The constant *Cst* can be determined by the boundary condition $y(\infty ,t)=0$. This gives:

15 $$2m\sqrt{Ct}{\int }_{0}^{\infty }\,{e}^{-{u}^{2}}du+Cst=0$$

With ${\int }_{0}^{\infty }\,{e}^{-{u}^{2}}du=\frac{\sqrt{\pi }}{2}$, one obtains: $Cst=-\,2m\sqrt{Ct}\frac{\sqrt{\pi }}{2}$ and equation (15) can be written:

$$y=2m\sqrt{Ct}{\int }_{0}^{\frac{x}{2\sqrt{Ct}}}\,{e}^{-{u}^{2}}du-2m\sqrt{Ct}\frac{\sqrt{\pi }}{2}=-\,m\sqrt{\pi Ct}[1-\frac{2}{\sqrt{\pi }}{\int }_{0}^{\frac{x}{2\sqrt{Ct}}}\,{e}^{-{u}^{2}}du]$$

In conclusion, the solution of approximated Mullins problem will be written as:

16 $$y(x,t)=-\,m\sqrt{\pi Ct}[1-\frac{2}{\sqrt{\pi }}{\int }_{0}^{\frac{x}{2\sqrt{Ct}}}\,{e}^{-{u}^{2}}du]$$

In conclusion for this part, the Mullins solution of approximated equation supposing |*y*′| ≪ 1, is given by:

17 $$\{\begin{array}{c}y(x,t)=-\,m\sqrt{\pi Ct}\,erfc(\frac{x}{2\sqrt{Ct}})\\ y{\prime} (u)=2m\sqrt{Ct}{e}^{-{u}^{2}},\,{\rm{with}}\,{\rm{u}}=\frac{x}{2\sqrt{Ct}}\end{array}$$

where erfc is the complementary error function.

The derivative *y*′ is given as a function of *x* and *t* by the following equation:

18 $$y{\prime} (x,t)=m\,{e}^{-\frac{{x}^{2}}{4Ct}}$$

However, knowing that $(0,t)=-\,{\varepsilon }_{0}$, one deduces the value of the groove depth:

19 $${\varepsilon }_{0}(t)=m\sqrt{\pi Ct}=tan\theta \sqrt{\pi Ct}$$

## Results Obtained by Using Mullins Approximation

**Figure 5:**
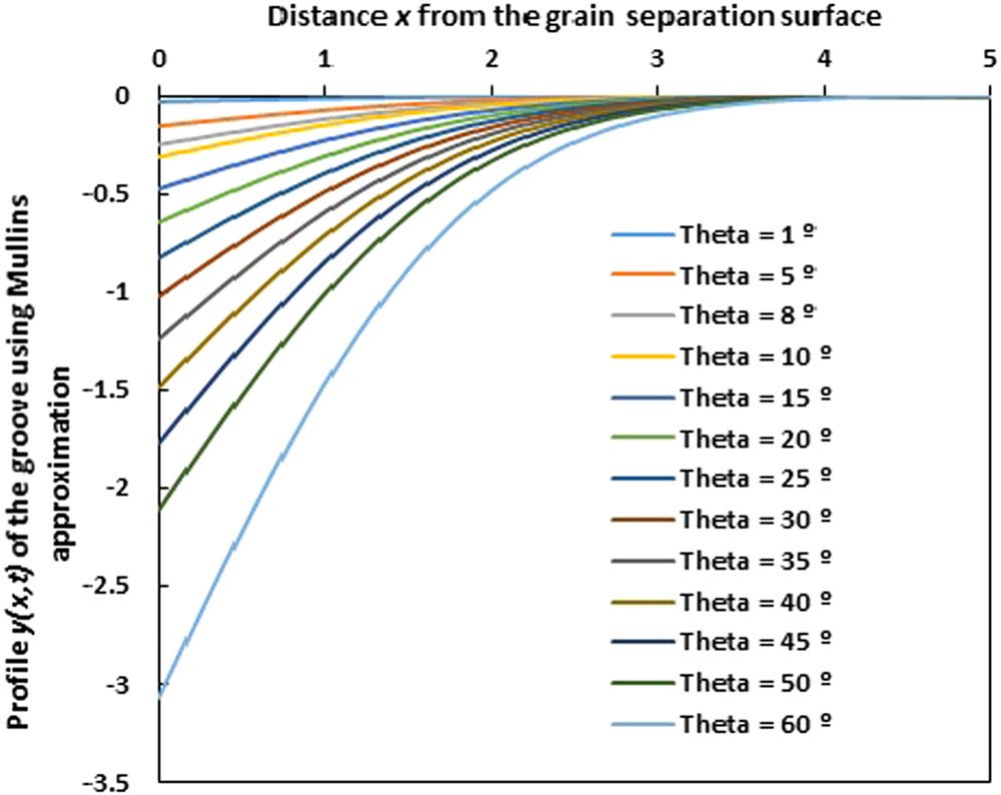
Evolution of the profile *y*(*x*, *t*) of the grain groove of Mullins approximation as a function of the distance *x* from the grain separation surface for various groove angles *θ* from 1° to 60°. (*Ct* = 1).

The approximation |*y*′| ≪ 1 used by Mullins in the case of evaporation and condensation in case of the grain boundary grooving in polycrystalline thin films is well described by equations (17). By taking *Ct* = 1 (value proposed and used by Mullins^10^), we obtain the Fig. 5 showing the variation of the profile *y*(*x*, *t*) of the grain groove of Mullins approximation as a function of the distance *x* from the grain separation surface for various angles *θ*. It is obvious shown that when the groove angle *θ* increases, the slope *m* at *x* = 0 increases and this increases the groove depth *ε*_0_ as proved by Fig. 5.

**Figure 6:**
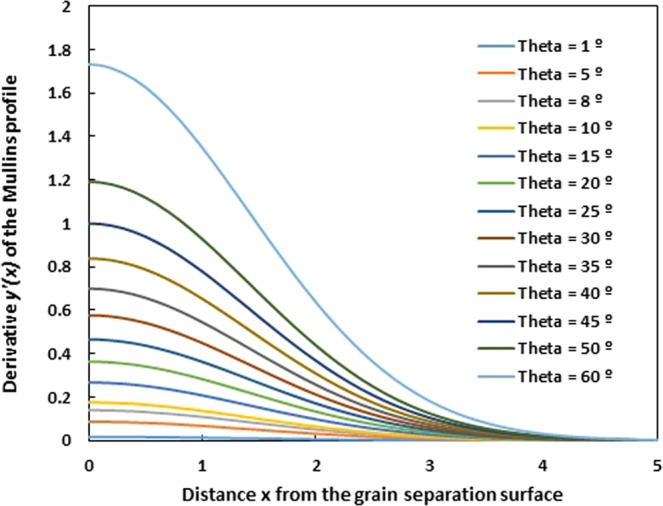
Evolution of the derivative *y*′ (*x*) of the grain groove profile of Mullins approximation as a function of the distance x from the grain separation surface for various groove angles *θ* from 1° to 60°.

On Fig. 6, we plotted the variations of the derivative *y*′ (*x*) of the grain groove profile for Mullins approximation as a function of the distance *x* from the grain separation surface for various angles *θ*. This derivative *y*′ strongly depends on the groove angle. The smaller the groove angle is, the smaller the derivative *y*′ is. For *θ* = 1°, all derivatives *y*′ (*x*) can be neglected relatively to 1 for all *x* values. In the nonrealistic case for *θ* ≤ 5°, the Mullins approximation can be applied and the solution can describe the profile of the grain groove in the case of evaporation-condensation.

It is clearly shown on Fig. 6 that for a groove angle and greater than 8°, all derivative values *y*′ (*x*) are greater than 0.15 whatever the *x* value and become greater than 1 when *θ* > 25°. In all cases, the Mullins approximation cannot be applied when the groove angle *θ* > 8° and the Mullins condition |*y*′| ≪ 1 becomes invalid in such cases.

Figure 6 clearly shows the non-validity of the Mullins hypothesis that supposed |y′| ≪ 1. The error percentage is higher than 20% from a groove angle *θ* exceeding 10°; the error dramatically increases to 35% for *θ* = 15° and exceeds 100% since *θ* = 30°. This proves that the Mullins approximation cannot be justified after *θ* = 7° and all results of the literature based on the condition |y′| ≪ 1 are experimentally false.

On the other hand, Mullins considered the condition of Mullins of the small slope approximation:

20 $$y\prime\prime\prime (0,t)=0$$

Even this condition proposed by Mullins is not in general satisfied.

To prove that this condition is not justified in this case, calculate the third derivative $y\prime\prime\prime (x,t)$ using the Mullins approximation:

21 $$y\prime\prime\prime (x,t)=-\,2m[1-\frac{{x}^{2}}{2Ct}]{e}^{-\frac{{x}^{2}}{4Ct}}$$

When applying the condition (20) and taking *Ct* = 1 as Mullins did it in^10^, we find:

22 $$y\prime\prime\prime (0,t)=-\,2m$$

Therefore, $y\prime\prime\prime (0,t)\ne 0$ and the condition (20) is not satisfied and it strongly depends on the slope *m* at the origin. However, if *m* > 0.05 or the angle *θ* > 3°, then $|y\prime\prime\prime (0,t)| > 0.10$. Therefore, the Mullins approximation is only satisfied if *θ* < 3°. This last case is not a realistic one.

## Solution of Broadbridge and Fujita

Broadbridge^18^ gave an implicit solution of the general Mullins problem given by equation (2) by applying the Fujita results^22–24^. Broadbridge replaced *C*(*T*) in equation (2) by a constant *D*(0) and wrote the following form:

23 $$\frac{\partial y}{\partial t}=D(0)\frac{y{\prime \prime} (x)}{(1+y{(x)}^{2})}$$

By using the following transformation:

24 $${\rm{\Theta }}={C}_{0}^{-1}y{\prime} ,\,{\rm{y}}={C}_{0}{\int }_{\infty }^{x}\,{\rm{\Theta }}(x,t)dx$$

By taking $D({\rm{\Theta }})=f({C}_{0}{\rm{\Theta }})$, Broadbridge^18^ deduced, with the same boundary conditions of Mullins problem, the following equation:

25 $$\frac{\partial {\rm{\Theta }}}{\partial t}=\frac{\partial }{\partial t}[D({\rm{\Theta }})\frac{\partial {\rm{\Theta }}}{\partial x}]$$

*D*(Θ) is given by:

26 $$D({\rm{\Theta }})=\frac{D(0)}{1+{C}_{0}^{2}{{\rm{\Theta }}}^{2}}$$

Broadbridge^18^ proposed the equation *ρ* = *g*(*η*), with:

27 $$\rho =\frac{1}{2}y{[D(0)t]}^{-1/2},\,\eta =\frac{1}{2}x{[D(0)t]}^{-1/2}$$

To deduce the following ordinary differential equation:

28 $$2[\rho -\eta \frac{\partial {\rm{\rho }}}{\partial \eta }]=\frac{1}{1+{(\frac{\partial \rho }{\partial \eta })}^{2}}\frac{{\partial }^{2}{\rm{\rho }}}{\partial {\eta }^{2}}$$

With the classical boundary conditions.

Based on the solution of Fujita^22–24^ that introduced a variable change, *ψ*, such as:

29 $$\frac{\partial {\rm{\psi }}}{\partial {\rm{\Theta }}}=2\eta $$

Satisfying: $$\{\begin{array}{c}\frac{\partial {\rm{\psi }}}{\partial {\rm{\Theta }}}=0\,at\,{\rm{\Theta }}=1\\ \psi \to 0\,as\,{\rm{\Theta }}\to 0\end{array}$$

Broadbridge^18^ obtained:

30 $$\rho ={C}_{0}[\eta {\rm{\Theta }}-\frac{1}{2}\psi ]$$

Notice that Θ = Θ = 1 for t > 0 and x = 0

Now, Fujita^22^ proposed the following variable changes:

31 $$\{\begin{array}{c}z=\frac{{\rm{\Theta }}}{{{\rm{\Theta }}}_{0}}={\rm{\Theta }}\,\\ \varphi ={C}_{0}\sqrt{\psi }\end{array}$$

and he obtained:

32 $$\frac{{d}^{2}\varphi }{d{z}^{2}}=-\,\frac{2}{(1+{z}^{2})\varphi }$$

Putting:

33 $$U=\frac{\sqrt{2}\varphi }{1+{z}^{2}}$$

and integrating equation (32), Fujita deduced:

34 $$(1+{z}^{2})\frac{dU}{dz}=\pm {(\lambda -{U}^{2}-8lnU)}^{1/2}$$

with *λ* an integration constant

Fujita^22^ Denoted by U_*_ the value of *U* determined by equation

35 $$\lambda -{U}_{\ast }^{2}-8\,ln\,{U}_{\ast }=0\,{\rm{for}}\,z={{\rm{z}}}_{\ast }={{\rm{\Theta }}}_{\ast }$$

Putting

36 $$\theta =\frac{U}{{U}_{\ast }},\,{\epsilon }=\frac{8}{{U}_{\ast }^{2}},\,{\rm{F}}(\theta ,{\epsilon })={C}_{0}{\int }_{0}^{\theta }\,{(1-{q}^{2}-8lnq)}^{-1/2}dq$$

Fujita^22^ then gave an implicit solution necessitating numerical calculations.

Broadbridge^18^ applied Fujita’s results to Mullins problem and obtained:

37 $${\rm{\Theta }}={C}_{0}^{-1}tan\,{\rm{F}}(\theta ,{\epsilon });\,0\le {\rm{\Theta }}\le {{\rm{\Theta }}}_{\ast }$$

38 $${\rm{\Theta }}={C}_{0}^{-1}tan[ta{n}^{-1}{C}_{0}-{\rm{F}}(\theta ,{\epsilon })+{\rm{F}}({\theta }_{m},{\epsilon })];\,{{\rm{\Theta }}}_{\ast }\le {\rm{\Theta }}\le 1$$

and

39 $${\rm{\psi }}=2{{\epsilon }}^{-1/2}{C}_{0}^{-1}sec\,[{\rm{F}}(\theta ,{\epsilon })];\,0\le {\rm{\Theta }}\le {{\rm{\Theta }}}_{\ast }$$

40 $${\rm{\psi }}=2{{\epsilon }}^{-1/2}{C}_{0}^{-1}sec[ta{n}^{-1}{C}_{0}-{\rm{F}}(\theta ,{\epsilon })+{\rm{F}}({\theta }_{m},{\epsilon })];{{\rm{\Theta }}}_{\ast }\le {\rm{\Theta }}\le 1$$

with

41 $${\rm{F}}(\theta ,{\epsilon })={C}_{0}{\int }_{0}^{si{n}^{-1}\theta }\,{(1-\frac{{\epsilon }ln\phi }{co{s}^{2}\phi })}^{-1/2}d\phi $$

The parameters Θ_*_, ${\epsilon }$ and *θ*_*m*_ are well defined by equations (22)–(24) of Broadbridge^18^ and can be calculated by numerical integration.

The final solution of Fujita is given here by the following equations:

42 $$\eta =\frac{{{\epsilon }}^{-1/2}}{\sqrt{1+{{\rm{\Theta }}}^{2}}}[\theta {\rm{\Theta }}+{(1-{\theta }^{2}-{\epsilon }ln\theta )}^{1/2}];0\le {\rm{\Theta }}\le {{\rm{\Theta }}}_{\ast }$$

43 $$\eta =\frac{{{\epsilon }}^{-1/2}}{\sqrt{1+{{\rm{\Theta }}}^{2}}}[\theta {\rm{\Theta }}-{(1-{\theta }^{2}-{\epsilon }ln\theta )}^{1/2}];\,{{\rm{\Theta }}}_{\ast }\le {\rm{\Theta }}\le 1$$

Equations (42) and (43) do not allow to obtain an explicit solution y(*x*, *t*) of Mullins problem. Therefore, it will be very difficult, in practice, to use these results. All these reasons lead us to reconsider the evaporation-condensation problem by proposing a new mathematical method taking into account the general equation without neglecting the first derivative *y*′(*x*). New expressions easier to be used were obtained. The new solution consisting in the correction of Mullins solution is presented in the following section.

## New Resolution of the General Case

The general equation of the evaporation-condensation problem is given by:

2 $$\frac{\partial y}{\partial t}=C(T)\frac{y{\prime \prime} (x)}{(1+y{\prime} {(x)}^{2})}$$

Using the same notations given above, one writes:

44 $$y{\prime \prime} (u)+2u\frac{1}{4Ct}y{\prime} {(u)}^{3}+2uy{\prime} (u)=0$$

and the second order differential equation is then given by the expression (45):

45 $$y{\prime \prime} (u)=-\,2uy{\prime} (u)[1+\frac{1}{4Ct}y{\prime} {(u)}^{2}]$$

Equation (45) can be written as:

46 $$\frac{dy{\prime} }{y{\prime} [1+\frac{1}{4Ct}y{{\prime} }^{2}]}=-\,2udu$$

${\rm{Putting}}=\frac{1}{4Ct}$, one obtains after decomposition into simple elements the following differential equation:

47 $$[\frac{1}{y{\prime} }-\frac{\beta y{\prime} }{(1+\beta y{{\prime} }^{2})}]dy{\prime} =-\,2udu$$

The integration of equation (47) will give:

48 $$ln[\frac{y{\prime} }{\sqrt{1+\beta {y}^{{\prime} 2}}}]=-\,{u}^{2}+Cst$$

Knowing that $y{\prime} (u)=\frac{\partial y}{\partial x}\frac{\partial x}{\partial u}=2\sqrt{Ct}\frac{\partial y}{\partial x}$ and using the boundary condition $y{\prime} (u=0)=2m\sqrt{Ct}$, one obtains:

49 $$Cst=ln[\frac{2m\sqrt{Ct}}{\sqrt{1+4\beta Ct{m}^{2}}}]=ln[\frac{2m\sqrt{Ct}}{\sqrt{1+{m}^{2}}}]$$

Therefore, the calculations lead to the first order differential equation (50):

50 $$\frac{y{\prime} }{\sqrt{1+\beta y{{\prime} }^{2}}}=\frac{2m\sqrt{Ct}}{\sqrt{1+{m}^{2}}}{e}^{-{u}^{2}}$$

By putting $\alpha =\frac{2m\sqrt{Ct}}{\sqrt{1+{m}^{2}}}$ and replacing it in equation (50), one obtains:

51 $$\frac{y{{\prime} }^{2}}{1+\beta {y}^{{\prime} 2}}={\alpha }^{2}{e}^{-2{u}^{2}}$$

This leads to the following equation: $y{\prime} (u)=+\,\frac{\alpha }{\sqrt{{e}^{2{u}^{2}}-\beta {\alpha }^{2}}}$. The variables *y* and *u* can be separated:

52 $$dy=+\,\frac{\alpha }{\sqrt{{e}^{2{u}^{2}}-\beta {\alpha }^{2}}}du$$

with:

$$\{\begin{array}{c}m=tan\theta \\ \alpha =2\sqrt{Ct}\,\sin \,\theta \\ 4\beta Ct=1\end{array}$$

One can write the first derivative *y*′(*u*) given by equation (53):

53 $$y{\prime} (u)=+\,\frac{\alpha }{\sqrt{{e}^{2{u}^{2}}-si{n}^{2}\theta }}$$

as a function of *x* and *t*, one writes$y{\prime} (x,t)=+\,\frac{\sin \,\theta }{\sqrt{{e}^{{x}^{2}/(2Ct)}-si{n}^{2}\theta }}$, and the integration of equation (53) leads to:

54 $$y(u)-y(0)={\int }_{0}^{u}\,\frac{\alpha }{\sqrt{{e}^{2{u}^{2}}-si{n}^{2}\theta }}du$$

Knowing that (0) = −*ε*_0_, where *ε*_0_ is the depth of the groove, we obtain equation (55):

55 $$y(u)={\int }_{0}^{u}\,\frac{\alpha }{\sqrt{{e}^{2{u}^{2}}-si{n}^{2}\theta }}du-{\varepsilon }_{0}$$

Using the boundary condition:${\mathrm{lim}}_{u\to \infty }y(u)=0$ and equation (55) one obtains the following equation:

56 $${\varepsilon }_{0}={\int }_{0}^{\infty }\,\frac{\alpha }{\sqrt{{e}^{2{u}^{2}}-si{n}^{2}\theta }}du$$

Therefore, equation (55) can be written as:

57 $$y(u)={\int }_{0}^{u}\,\frac{\alpha }{\sqrt{{e}^{2{u}^{2}}-si{n}^{2}\theta }}du-{\int }_{0}^{\infty }\frac{\alpha }{\sqrt{{e}^{2{u}^{2}}-si{n}^{2}\theta }}du$$

By writing equation (57) as a function of *x* and *t*, one obtains:

58 $$y(x,t)={\int }_{0}^{\frac{x}{2\sqrt{Ct}}}\,\frac{sin\theta }{\sqrt{{e}^{{v}^{2}/(2Ct)}-si{n}^{2}\theta }}dv-{\int }_{0}^{\infty }\,\frac{sin\theta }{\sqrt{{e}^{{v}^{2}/(2Ct)}-si{n}^{2}\theta }}dv$$

or

59 $$y(x,t)={\int }_{\infty }^{x/2\sqrt{Ct}}\frac{sin\theta }{\sqrt{{e}^{{v}^{2}/(2Ct)}-si{n}^{2}\theta }}dv$$

**Figure 7:**
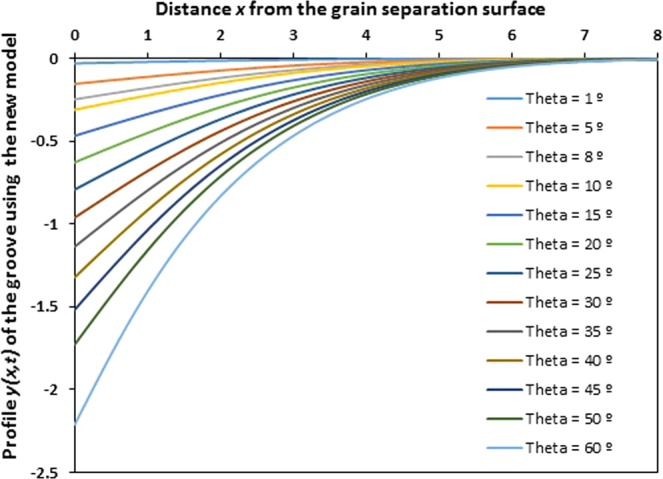
Profile *y*(*x*, *t*) of the grain groove of the new solution as a function of the distance *x* from the grain separation surface for various groove angles *θ*. (*Ct* = 1).

Equation (59) giving the new solution obviously shows the large difference with the Mullins solution. On Fig. 7, we represent the variations of the profile *y*(*x*, *t*) of the grain groove of the new solution as a function of the distance *x* from the grain separation surface for various angles *θ*, for a particular and specific case such as *Ct* = 1 (value used by Mullins) in order to have the same situation of Mullins and to be able to compare our new solution with that of Mullins approximation. All calculations were executed using Mathematica programme.

## Comparison Between Mullins Solution and the Exact Solution

The following expression gives the ratio of Mullins solution *y*_*Mullins*_ on the exact solution *y*_*exact*_:

60 $$\frac{{y}_{Mullins}}{{y}_{exact}}=\frac{ \mbox{-} \tan \,\theta \sqrt{\pi Ct}erfc(\frac{x}{2\sqrt{Ct}})}{{\int }_{\infty }^{x/2\sqrt{Ct}}\,\frac{sin\theta }{\sqrt{{e}^{{v}^{2}/(2Ct)}-si{n}^{2}\theta }}dv}$$

**Figure 8:**
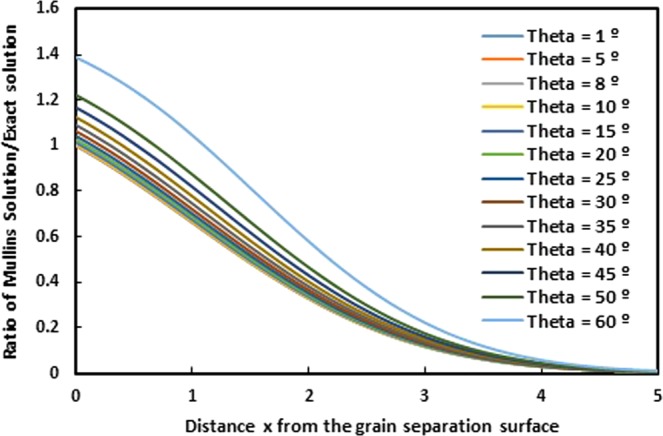
Evolution of the ratio (*y*_*Mullins*_/*y*_*exact*_) of Mullins solution on the exact solution for the grain groove profile as a function of the distance *x* from the grain separation surface for various angles *θ*. (*Ct* = 1).

On Fig. 8, we represent the variations of the ratio (*y*_*Mullins*_/*y*_*exact*_) as a function of the distance *x* from the grain separation surface for various groove angles *θ*. The obtained curves clearly show an important deviation between the two solutions for all values of groove angle *θ*.

**Figure 9:**
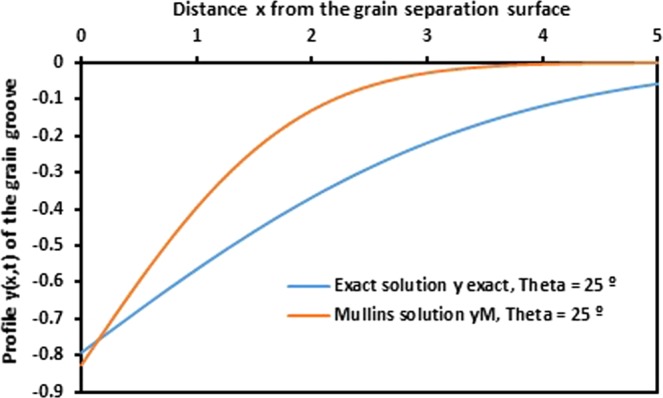
Evolution of the profile *y* (*x*) of the grain groove for Mullins approximation and the exact solution as a function of the distance *x* from the grain separation surface for *θ* = 25°. (*Ct* = 1).

**Figure 10:**
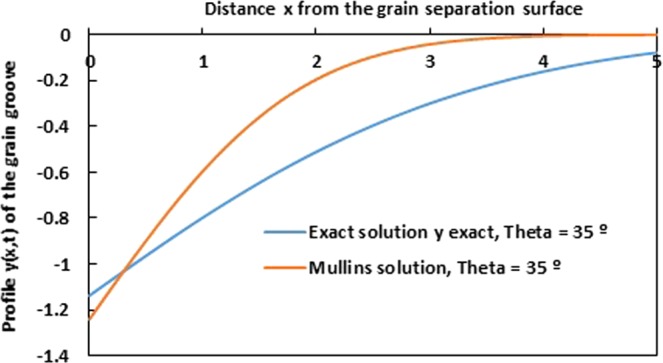
Evolution of the profile *y* (*x*) of the grain groove for Mullins approximation and the exact solution as a function of the distance *x* from the grain separation surface for *θ* = 35°. (*Ct* = 1).

As example, we draw on Figs 9 and 10, the evolution of the profile *y* (*x*) of the grain groove for Mullins approximation and the exact solution as a function of the distance *x* from the grain separation surface, respectively for *θ* = 25° and 35°. The two obtained curves on Figs 9 and 10 show an important difference between the two cases. Mullins solution is really so far from the exact solution.

**Figure 11:**
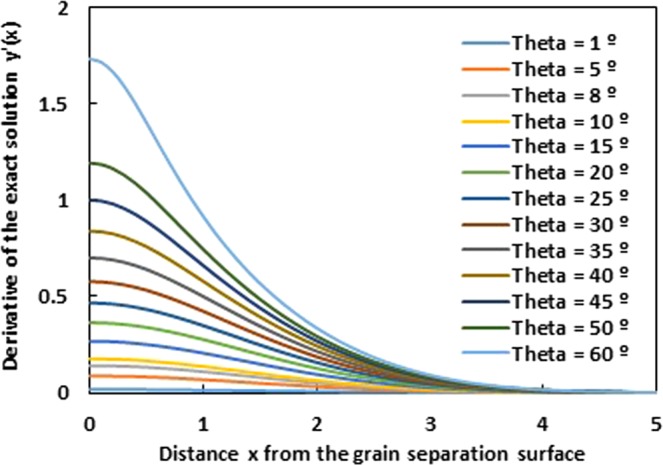
Evolution of the derivative *y*′ (*x*) of the grain groove profile of the exact solution as a function of the distance x from the grain separation surface for various groove angles *θ*. (*Ct* = 1).

The calculation of the exact derivative *y*′ (*x*) of the grain groove profile obtained by the exact solution given by equation (53) for various groove angles *θ* is represented on Fig. 11. Here, we didn’t suppose any condition on the value of the first derivative of the grain groove profile.

Now, by expressing the ratio of the Mullins derivative *y*′_*Mullins*_ and the exact derivative *y*′_*exact*_, we obtain the following expression:

61 $$\frac{y{{\prime} }_{Mullins}}{y{{\prime} }_{exact}}=\frac{\sqrt{1-{e}^{-\frac{{x}^{2}}{2Ct}}si{n}^{2}\theta }}{cos\theta }\,$$

**Figure 12:**
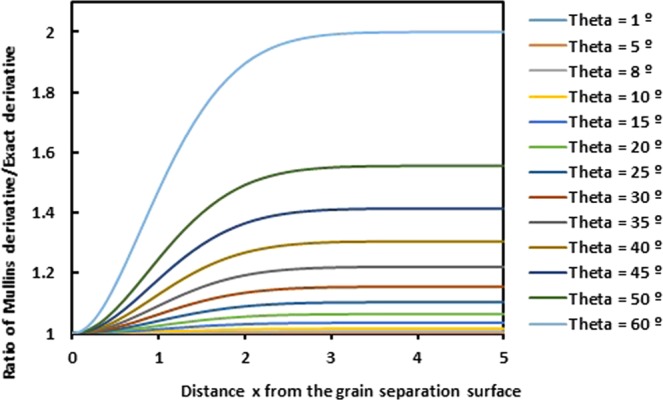
Evolution of the ratio of Mullins derivative on the exact derivative of the grain groove profile as a function of the distance *x* from the grain separation surface for different values of groove angle *θ*. (*Ct* = 1).

In order to compare between the two derivatives of Mullins and exact solutions, we draw on the Fig. 12, the evolution of the ratio of Mullins derivative of the grain groove profile on the exact derivative as a function of the distance *x* from the grain separation surface for different groove angles *θ*. The two obtained curves show an extreme deviation when the distance *x* increases.

Even for the derivatives *y*′_*Mullins*_ and *y*′_*exact*_, there is an important difference between the two derivatives and the deviation can reach 200% putting in defeat the Mullins model.

## Calculation of the Groove Depth

Equation (56) can be written as:

62 $${\varepsilon }_{0}={\int }_{0}^{\infty }\,\frac{\alpha {e}^{-{u}^{2}}}{\sqrt{1-{e}^{-2{u}^{2}}si{n}^{2}\theta }}du$$

Now, using Taylor series expansion for a radius of convergence equal to 1:

63 $${(1-x)}^{p}=1+\sum _{n=1}^{\infty }\,\frac{{(-1)}^{n}p(p-1)\ldots (p-n+1)}{n!}{x}^{n}$$

For *p* = −1/2, equation (62) becomes for |*X*| < 1:

64 $${(1-X)}^{-1/2}=1+\sum _{n=1}^{\infty }\,\frac{(2n)!}{{(n!)}^{2}{2}^{2n}}{X}^{n}$$

Applying this formula for $X={e}^{-2{u}^{2}}si{n}^{2}\theta  < 1$, one can write:

65 $${(1-{e}^{-2{u}^{2}}si{n}^{2}\theta )}^{-1/2}=1+\sum _{n=1}^{\infty }\,\frac{(2n)!}{{(n!)}^{2}{2}^{2n}}({e}^{-2n{u}^{2}}si{n}^{2n}\theta )$$

Equation (61) can be then written as:

66 $${\varepsilon }_{0}=\alpha {\int }_{0}^{\infty }\,[{e}^{-{u}^{2}}+\sum _{n=1}^{\infty }\,\frac{(2n)!}{{(n!)}^{2}{2}^{2n}}({e}^{-3n{u}^{2}}si{n}^{2n}\theta )]du$$

By permuting between integral and summation signs in equation (66) because of the obvious convergence of the series and knowing that ${\int }_{0}^{\infty }\,{e}^{-{u}^{2}}du=\frac{\sqrt{\pi }}{2}$, one obtains:

67 $${\varepsilon }_{0}=\frac{\alpha \sqrt{\pi }}{2}[1+\sum _{n=1}^{\infty }\,\frac{(2n)!}{{(n!)}^{2}{2}^{2n}\sqrt{3n}}si{n}^{2n}\theta ]$$

Using $\alpha =2\sqrt{Ct}\,\sin \,\theta$, the following expression for *ε*_0_ will be obtained:

68 $${\varepsilon }_{0}=\sqrt{\pi Ct}\,\sin \,\theta [1+\sum _{n=1}^{\infty }\,\frac{(2n)!}{{(n!)}^{2}{2}^{2n}\sqrt{3n}}si{n}^{2n}\theta ]$$

**Figure 13:**
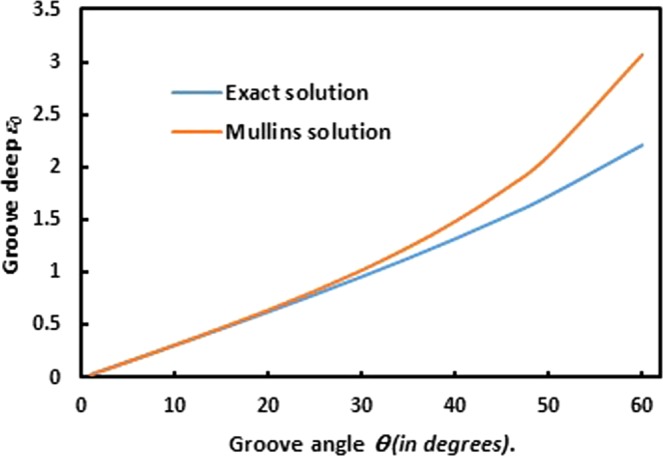
Evolution of the Groove deep *ε*_0_ as a function the groove angle *θ* in two cases of Mullins solution and the exact solution.

Equation (68) clearly shows that the groove depth *ε*_0_ strongly depends on the groove angle *θ* the coefficient *C* and the time *t*. The comparison between Mullins and exact solutions leads to draw the following Fig. 13:

Figure 13 shows an important deviation of Mullins groove deep relative to the exact groove deep for *θ* larger than 25 °. On the other hand, the groove deep increases when the groove angle increases thus showing a strong dependency of these two parameters.

## Analytical Solution

In this section, we propose to give an analytical solution of the general case. Equation (58) can be rearranged as:

69 $$y(u)={\int }_{0}^{u}\frac{\alpha {e}^{-{u}^{2}}}{\sqrt{1-{e}^{-2{u}^{2}}si{n}^{2}\theta }}du-{\varepsilon }_{0}$$

Following the same previous method for the integration of equation (69), one writes:

70 $$y(u)=\alpha {\int }_{0}^{u}\,[{e}^{-{v}^{2}}+\sum _{n=1}^{\infty }\,\frac{(2n)!}{{(n!)}^{2}{2}^{2n}}({e}^{-3n{v}^{2}}si{n}^{2n}\theta )]dv-{\varepsilon }_{0}$$

By permuting between integral and summation signs, equation (70) can be written as:

71 $$y(u)=\alpha [{\int }_{0}^{u}\,{e}^{-{v}^{2}}dv+\sum _{n=1}^{\infty }\,\frac{(2n)!}{{(n!)}^{2}{2}^{2n}}si{n}^{2n}\theta {\int }_{0}^{u}\,{e}^{-3n{v}^{2}}dv]-{\varepsilon }_{0}$$

If one uses the complementary error function erfc:

$${\int }_{0}^{u}\,{e}^{-{v}^{2}}dv=\frac{\sqrt{\pi }}{2}[1-erfc(u)],$$

$${\int }_{0}^{u}\,{e}^{-3n{v}^{2}}dv=\frac{\sqrt{\pi }}{2\sqrt{3n}}[1-erfc(\sqrt{3n}u)]$$

One finds:

72 $$y(u)=\frac{\alpha \sqrt{\pi }}{2}[1-erfc(u)+\sum _{n=1}^{\infty }\,\frac{(2n)!}{{(n!)}^{2}{2}^{2n}\sqrt{3n}}si{n}^{2n}\theta (1-erfc(\sqrt{3n}u))]-{\varepsilon }_{0}$$

with $=\frac{x}{2\sqrt{Ct}}$, and $\alpha =2\sqrt{Ct}\,\sin \,\theta$, the final solution can be written as a function of *x* and *t* as:

73 $$y(x,t)=\sqrt{\pi Ct}sin\theta [1-erfc(\frac{x}{2\sqrt{Ct}})+\sum _{n=1}^{\infty }\,\frac{(2n)!}{{(n!)}^{2}{2}^{2n}\sqrt{3n}}si{n}^{2n}\theta (1-erfc(\frac{x\sqrt{3n}}{2\sqrt{Ct}}))]-{\varepsilon }_{0}$$

Replacing *ε*_0_ by its value from equation (72), one obtains the final solution given by equation (78):

74 $$y(x,t)=-\,\sqrt{\pi Ct}sin\theta [erfc(\frac{x}{2\sqrt{Ct}})+\sum _{n=1}^{\infty }\,\frac{(2n)!}{{(n!)}^{2}{2}^{2n}\sqrt{3n}}si{n}^{2n}\theta (erfc(\frac{x\sqrt{3n}}{2\sqrt{Ct}}))]$$

## Discussion of the Third Derivative Condition and Comments on the Constant *C*

### On the third derivative

The Mullins approximation lead us to determine the third derivative

21 $$y\prime\prime\prime (x,t)=-\,2m[1-\frac{{x}^{2}}{2Ct}]{e}^{-\frac{{x}^{2}}{4Ct}}$$

The third Mullins condition $y\prime\prime\prime (0,t)=0$ relative to the small slope approximation is not satisfied when using the Mullins approximation, because equation (21) leads to $y\prime\prime\prime (0,t)=-\,2m\ne 0$

Now, the second derivative *y*″(*u*) from our exact solution is recalled here:

45 $$y{\prime \prime} (u)=-\,2uy{\prime} (u)[1+\beta y{\prime} {(u)}^{2}]$$

The calculation of the third derivative leads to the following expression:

75 $$y\prime\prime\prime (u)=2y{\prime} (u)(1+\beta y{\prime} {(u)}^{2})[2{u}^{2}(1+3\beta y{\prime} {(u)}^{2})-1]$$

Knowing that $\,{\prime} (u)=2\sqrt{Ct}y{\prime} (x,t)$, $y\prime\prime\prime (u)=2\sqrt{Ct}y\prime\prime\prime (x,t)$, $u=\frac{x}{2\sqrt{Ct}}$ and $4\beta Ct=1$, one obtains:

76 $$y\prime\prime\prime (x,t)=2y{\prime} (x,t)(1+y{\prime} {(x,t)}^{2})[\frac{\,{x}^{2}}{2Ct}(1+3y{\prime} {(x,t)}^{2})-1]$$

where the first derivative is given by: $y{\prime} (x,t)=+\,\frac{\sin \,\theta }{\sqrt{{e}^{{x}^{2}/(2Ct)}-si{n}^{2}\theta }}$ and $\,{\prime} (0,t)=tan\theta =m$. This leads to the value of $y\prime\prime\prime (0,t)$:

77 $$y\prime\prime\prime (0,t)=-\,2m(1+{m}^{2})$$

Therefore, we can evaluate the percentage error relative to the use of the Mullins approximation:

78 $$\frac{{[y\prime\prime\prime (0,t)]}_{Exact}-{[y\prime\prime\prime (0,t)]}_{Mullins}}{{[y\prime\prime\prime (0,t)]}_{Exact}}=100\frac{m}{1+{m}^{2}}$$

If *m* > 0.10, the groove angle *θ* > 6°.

Again, we proved the non-validity of the Mullins approximation.

### On the constant C and practical cases of metals

In order to study the effect of the parameter *C* or *Ct* on the validity of Mullins approximation that supposed the condition |*y*′ (*x*)| ≪ 1 for any x whatever, we calculated below the values of the parameter *C* for various metals as a function of temperature *T*. To do this, recall the relation giving C as a function of the different characteristics of metals:

3 $$C(T)=\frac{{P}_{0}(T)\gamma {\omega }^{2}}{{(2\pi m)}^{1/2}{(kT)}^{3/2}}$$

The vapor pressure *P*_0_(*T*) of metals can be calculated by using the following general equation^39^:

78 $$\mathrm{log}\,{P}_{0}(atm)=a+\frac{b}{T}+c\,\mathrm{log}\,T+\frac{d}{{10}^{3}}T$$

where *γ* the constants a, b, c and d are given for different metals on Table 1:

**Table 1:** Values of a, b, c and d for different metals for valid temperature range.

Metal	a	b	c	d	Temperature range (K)
Co	6.488	−20578	0	0	1768.2–2150 K
Zn (solid)	8.435	−6923	−0.7523	0	298–692.6 K
Ga	3.624	−13829	0.7579	−0.3141	302.96–1600 K
Ti	16.37	−25229	−2.6574	0	1941.2–2400 K
Tl	8.628	−9383	−1.0086	0	577.2–1100 K
Li	8.409	−8320	−1.0255	0	453.7–1000 K
Au	10.298	−18898	−1.2222	0	1337.2–2050 K
Cu	11.209	−17427	−1.4742	0	1358.2–1850 K
Al	10.578	−16946	−1.313	0	933.5–1800 K
Cs	8.232	−4062	−1.3359	0	302.96–550 K
Sr	4.809	−8385	0.415	−0.597	298–1050.2 K
Mg	8.489	−7813	−0.8253	0	298–923.2 K
Zn (liquid)	5.378	−6286	0	0	692.6–750 K

Equation (78) and Table 1 allowed to calculate the vapor pressures of different metals. The different physicochemical characteristics of the metals are presented on Table 2.

**Table 2:** Characteristics of tested metals such as melting point: *T*_*MP*_ (K), boiling point: *T*_*BP*_ (K), temperature of metal: *T* (K), vapor pressure at T: *P*_0_ (Pa), molar mass: *M* (g/mol), surface energy of metal: *γ* (J/m^2^)^40^ and atomic volume: *ω* (m^3^).

Metal	*T*_*MP*_ (K)	*T*_*BP*_ (K)	*T* (K)	*P*_0_ (Pa)	*M* (g/mol)	*γ* (J/m^2^)	*ω* (m^3^)
Co	1768.2	3143.2	2120	303.04	58.933	2.536	1.11 × 10^−29^
Zn (solid)	692.6	980.2	688	86.67	65.38	0.992	1.53 × 10^−29^
Ga	302.96	2673.2	1570	278.52	69.723	0.991	1.96 × 10^−29^
Ti	1941.2	3560.2	2370	286.35	47.867	2.045	1.77 × 10^−29^
Tl	577.2	1746.2	1070	318.79	204.383	0.639	2.86 × 10^−29^
Li	453.7	1603.2	970	294.34	6.941	0.524	2.18 × 10^−29^
Au	1337.2	2973.2	2300	4708.39	196.9666	1.503	1.69 × 10^−29^
Cu	1358.2	2835.2	2200	11490.38	63.546	1.808	1.18 × 10^−29^
Al	933.5	2743.2	2000	2956.96	26.9815	1.152	2.32 × 10^−29^
Cs	302.96	2673.2	530	425.19	132.905	0.095	1.18 × 10^−28^
Sr	1050.2	1655.2	1030	1008.65	87.62	0.415	5.60 × 10^−29^
Mg	923.2	1364.2	910	1449.73	24.305	0.773	2.32 × 10^−29^
Zn (liquid)	692.6	980.2	950	28923.82	65.38	0.992	1.53 × 10^−29^

Using the results given in Table 2 and equation (3), we determined the values of *C* and the derivative *y*′ (*x*) of the various metals for the considered temperatures as a function of the distance *x*. The obtained results are presented on Table 3.

**Table 3:** Values of Mullins derivative *y*′ (*x*) versus x for the various metals.

Metal	*C* (μm^2^/s)	*Ct* (μm)	Values of approximated Mullins derivative *y*′ (*x*)				
			*x* = 0 μm	*x* = 0.01 μm	*x* = 0.1 μm	*x* = 0.5 μm	*x* = 1 μm
Co	2.42 × 10^−8^	0.0242	1	0.9990	0.9020	0.0789	–
Zn (solid)	2.63 × 10^−8^	0.0263	1	0.9990	0.9092	0.0926	–
Ga	3.89 × 10^−8^	0.0389	1	0.9994	0.9378	0.2009	0.0726
Ti	4.37 × 10^−8^	0.0437	1	0.9994	0.9444	0.2394	0.1427
Tl	6.34 × 10^−8^	0.0634	1	0.9996	0.9613	0.3730	0.1821
Li	1.75 × 10^−7^	0.1750	1	0.9999	0.9858	0.6996	0.2396
Au	2.50 × 10^−7^	0.2505	1	0.9999	0.9901	0.7792	0.3685
Cu	6.70 × 10^−7^	0.6704	1	1.0000	0.9963	0.9110	0.6887
Al	7.54 × 10^−7^	0.7535	1	1.0000	0.9967	0.9204	0.7176
Cs	7.64 × 10^−7^	0.7638	1	1.0000	0.9967	0.9214	0.7209
Sr	8.08 × 10^−7^	0.8081	1	1.0000	0.9969	0.9256	0.7339
Mg	8.51 × 10^−7^	0.8510	1	1.0000	0.9971	0.9292	0.7454
Zn (liquid)	5.40 × 10^−6^	5.4015	1	1.0000	0.9995	0.9885	0.9548

Table 3 clearly shows for 12 metals that the parameter *C* depends on the metal nature and imposed by the temperature range. Results show once again that the derivative *y*′ (*x*) cannot be neglected in front of 1 for all x values. The Mullins approximation is false even when the parameter *C* decreases from 10^−6^ to 10^−8^ μm^2^/s and for many studied metals. We conclude that the Mullins case can be only applied for groove angle smaller than 6 °C, the committed error reaching in certain cases 100%. The approximated solution obtained by Mullins cannot then describe the physical reality of the grain groove problem in the case of evaporation/condensation.

## Conclusion

This New study gave an exact and explicit solution and another analytical solution of the grain boundary groove profile relative to the case of evaporation/condensation. The resolution of the partial differential equation governing this phenomenon gave new analytical expressions of the groove profile *y*(*x*, *t*), the derivative *y*′(*x*, *t*) and the groove deep *ε*_0_(θ).

$$\begin{array}{c}y(x,t)=-\,\sqrt{\pi Ct}sin\theta [erfc(\frac{x}{2\sqrt{Ct}})+\sum _{n=1}^{\infty }\,\frac{(2n)!}{{(n!)}^{2}{2}^{2n}\sqrt{3n}}si{n}^{2n}\theta (erfc(\frac{x\sqrt{3n}}{2\sqrt{Ct}}))]\\ y{\prime} (x,t)=+\,\frac{\sin \,\theta }{\sqrt{{e}^{\tfrac{{x}^{2}}{(2Ct)}}-si{n}^{2}\theta }}\,{\rm{and}}\,{\varepsilon }_{0}(\theta )=\sqrt{\pi Ct}\,\sin \,\theta [1+\sum _{n=1}^{\infty }\frac{(2n)!}{{(n!)}^{2}{2}^{2n}\sqrt{3n}}si{n}^{2n}\theta ]\end{array}$$

The general solution given by Broadbridge^18^, and Fujita^22^ is not explicit solution, very complicated to be used by the scientific community and needs many numerical calculations. The equations obtained by our new method are easier and can be used for many practical applications.

The comparison with Mullins solution proved that the results obtained by Mullins were false and our new solution corrected the gap caused by the Mullins theory. This theory was only valid for non-realistic case of a groove angle *θ* ≈ 1° to 6°. The Mullins’s hypothesis expressed by |*y*′| ≪ 1 is not satisfied even when C is very small. The experimental values of 12 chosen metals confirmed the non-validity of the Mullins approximation. Even the third Mullins derivative $y\prime\prime\prime (0,t)$ cannot be cancelled as supposed by the third Mullins hypothesis. Our new solution proved that the error percentage of Mullins solution reaches 100% for values of the groove angle *θ* less than 30°.

The new solution gave an important and useful analytical correlation between the groove profile and the groove deep with the groove angle.
